# The scope of the global history of science: an essay on James Poskett’s Horizons*

**DOI:** 10.1590/S0104-59702026000100031

**Published:** 2026-08-14

**Authors:** Jose Daniel Serrano-Juarez, Brian Becerra-Bressant, Marco Ornelas-Cruces, Nuria Silis Gutierrez-Gaytan, Diana Galvan, Lucia Granados-Riveros, Teresa Villegas-Lopez, María Alicia Villela-González

**Affiliations:** i Associate researcher, Institute of Geography/Universidad Nacional Autónoma de México. Mexico City – Mexico. josedanielserrano@filos.unam.mx; ii PhD student, Graduate Program in Biological Sciences/Faculty of Sciences/Universidad Nacional Autónoma de México. Mexico City – Mexico; iii PhD student, Graduate Program in Philosophy of Science/Universidad Nacional Autónoma de México. Mexico City – Mexico; iv Master of Science, University Seminar on History, Philosophy and Studies of Science and Medicine/Universidad Nacional Autónoma de México. Mexico City – Mexico.; v Master’s student, Graduate Program in Philosophy of Science/Universidad Nacional Autónoma de México. Mexico City – Mexico; vi Master’s student, Graduate Program in Philosophy of Science/Universidad Nacional Autónoma de México. Mexico City – Mexico; vii Master’s student, Graduate Program in Philosophy of Science/Universidad Nacional Autónoma de México. Mexico City – Mexico; viii Master of Science, Department of Evolutionary Biology/Faculty of Sciences/Universidad Nacional Autónoma de México. Mexico City – Mexico

**Keywords:** Global history of science, Historical methods and theory, Otherness, Globality, Knowledge circulation, Historia Global de la Ciencia, Teoría y métodos históricos, Alteridad, Globalidad, Circulación del conocimiento

## Abstract

This historiographic review poses a reflection on the exciting field of the global history of science, and its methods and theory. We use the reading of *Horizons: a global history of science*, by James Poskett, as an exercise to ponder the scope and limitations of this historiographic approach. The analysis focuses on the concepts of otherness, globality, and circulation, three major transversal and interrelated themes treated in the book. These issues come to light through the author’s narrative analysis, and we consider that any writing about global history should deeply reflect upon such themes.

## Resumen

Esta revisión historiográfica plantea una reflexión sobre el interesante campo de la historia global de la ciencia, así como sobre sus métodos y su teoría. Utilizamos la lectura de Horizontes: una historia global de la ciencia, de James Poskett, como ejercicio para reflexionar sobre el alcance y las limitaciones de este enfoque historiográfico. El análisis se centra en los conceptos de alteridad, globalidad y circulación, tres grandes temas transversales e interrelacionados que se tratan en el libro. Estas cuestiones salen a la luz a través del análisis narrativo del autor, y consideramos que cualquier escrito sobre historia global debería reflexionar profundamente sobre dichos temas.

In his recent *Horizons: a global history of science*, James Poskett (2022, p.1) takes his readers to different places and times, recounting the story of a selected set of scientific theories and instruments with a fluent prose style. He proposes a new narrative about the origins of modern science in which it is seen not so much as a product of European culture but rather as the result of a convergence of people and ideas from different cultures worldwide.

After obtaining his PhD in 2015 at Trinity College, University of Cambridge, James Poskett was an Adrian research fellow at Darwin College, also at Cambridge, between 2015 and 2017. In 2017 he joined the University of Warwick as assistant professor, being promoted to associate professor in 2021, and reader in the History of Science and Technology in 2024 (Poskett, 7 Oct. 2024).

In 2015, he began his career with research on the history of phrenology and mental medicine in the United Kingdom (Poskett, 2015a, 2015b, 2017), while gradually turning his attention to the international connections that underpinned some of nineteenth-century science (Poskett, 2017). Although present in his early work, Poskett more fully incorporated a focus on global history in his first book (Poskett, 2019). However, with the 2022 publication with Penguin of *Horizons*, which has won multiple awards from English-speaking institutions, he has gained worldwide acclaim. The book has already been translated into French, German, Dutch, Spanish, Portuguese, Italian, Romanian, Greek, Chinese, Japanese, and Korean (Poskett, 7 Oct. 2024).

This historiographic review analyzes the implicit methodology used by Poskett, as well as the concepts of otherness, globality, and circulation, three major transversal and interrelated themes treated in greater or lesser breadth in the eight chapters of the book. These concepts, which are revealed in an examination of the author’s narrative, are ones we believe any writing about global history should deeply reflect upon. From one perspective, we recognize the advantages of approaching the history of science or sciences in this way. We can also learn about the many and varied local histories through books that take a similar approach. Conversely, we also consider reading this book to be a self-reflective exercise on the scope and limitations of writing a global history of science.

This is because we, the authors, are a group of historians of science in Mexico who belong to one of the regions that, according to Poskett (2022, p.7), are “largely forgotten today.” We are currently pursuing graduate studies and are also involved in teaching activities, so we are interested in texts that can help us introduce students to the history of science. Indeed, the “Critical Reading Group” workshop^1^ – held in October and November 2023 at the Faculty of Sciences of the National Autonomous University of Mexico (Universidad Nacional Autónoma de México), in Mexico City – was the starting point of our regular meetings to discuss this book.

This is pertinent because we agree with Haraway (1988) that impersonal scientific writing masks the situation in which each author or, in our case, group of authors, is situated and understands the world, also in historical terms. Thus, we are aware that the reflections we offer are made possible, but at the same time are biased, by our geographical, historical, and professional situations. For this work, we studied Poskett’s book both individually and within the Critical Reading Group. The eight participants read the whole book, and then each focused on one or two chapters. Group discussions allowed both a comparative analysis of recurring topics and collaborative work with different perspectives that articulated the structure and content of the book. We have divided our paper into the following sections: Reception, Methodology, Otherness, Globality and Circulation, and Concluding remarks.

## Reception

*Horizons* has been enthusiastically reviewed in its original English and its many translations. This is in large part due not only to Poskett’s academic background, but to his interest in communicating the history of science to a broader audience. As many of the book’s reviewers have remarked, his commitment to dissemination is evident in the narrative strategies he uses to achieve a clear and enjoyable reading experience. As Luk (2024, p.398) points out, Poskett succeeds in representing the scientific legacy that took place outside the West with great skill and captivating anecdotes, making it accessible to all audiences. The book is largely held to challenge the conventional Eurocentric narrative of modern scientific progress and reveal the intricate global exchanges of culture, through which the author shatters the notion that modern science emerged exclusively in Western Europe (e.g., Luk, 2024, p.397).

The reviews maintain that, through the description of various case studies, Poskett adopts a novel methodology, the basis of which is the circulation of ideas, characters, practices and instruments (Van Damme, 2023). It is the movement of people across borders, and the subsequent encounters with diverse worldviews and cultural practices, that revitalize a stale history. Alongside Luk (2024, p.398), Yılmaz (2023, p.968) also holds that *Horizons* stands out thanks to the credit it gives to many non-Western cultures, challenging the idea of isolated, disinterested men with great minds whose ingenuity pushed the development of knowledge.

Almost all the reviews and commentaries consider Poskett’s latest book a masterpiece: a provocative work with a capacity to revolutionize our perception of the origins of modern science. Some reviewers go further still, predicting that the text is destined to become a landmark and reference in the historiography of science (Van Damme, 2023), whose originality is even compared to studies such as Shapin’s (1996) *Scientific revolution* (Arias, 2023). Thus, there is a large consensus of acclaim and fascination for the author, who is held as a paragon as to how the history of science ought to be reconstructed.

Many of Poskett’s Spanish-language reviews also agree with the English-language reviewers. As one of them states, the global history of modern science “is not born from the self-absorption of a handful of Europeans, but from the web of interactions between cultures that came hand in hand with modern military and commercial expansion”^2^ (Arias, 2023). By and large, all reviewers laud the appearance of “newly discovered” personalities who had thus far been totally eclipsed by the Eurocentric big-picture account. They now have a place in Poskett’s narrative as the substrate of the great Western heroes, geniuses, and canons.

However, the reception of *Horizons* has not been limited to academic circles, as it has had considerable acceptance in wider reading circuits. Some of its reviews on the internet are unreservedly eulogistic (e.g., Bycroft, 27 Mar. 2022). The vast number and origins of these online reviews from different regions of the world are witness to the wide reach and number of readers that the work has had. An overwhelming majority consider that Poskett is required reading for understanding the dynamics of a “true” global history. One example of this reception emphasizes that *Horizons* “offers a powerful argument that an honest and accurate account of the development of modern science must include from around the world” (Moses, 23 Feb. 2023). Another states that “*Horizons* is an excellent reference text and corrective, a solid kernel around which a new understanding of history can grow” (Chandran, 7 Nov. 2022).

That said, some reviewers question the supposed virtues of *Horizons*. Despite his overall praise of the book, Moses (23 Feb. 2023) also states that Poskett “does not question what counts as ‘modern science’,” that “it is only through interactions with Europeans that people from the rest of the world can be said to participate in science” and that only some parts of indigenous knowledge can be understood as scientific. Similarly, Cañizares-Esguerra (2022, p.467) highlights that, although Poskett gives examples of non-European science and relates science to colonialism, capitalism, or slavery, his work “remains firmly grounded in Eurocentric teleologies,” because it is based on “European markers of progress: Copernicanism, Newtonianism, Linnaean natural history, Maxwellian electromagnetism.” For his part, Zamora (6 June 2022) suggests a careful reading of the book given Poskett’s academic and geographical positioning, and he indicates that the author’s extensive bibliography includes only five works not originally written in English, all of which are by French authors.

All in all, even though we believe that the exercise of showing how modern science is the product of global and not merely European exchanges, circulations, and confrontations of knowledge and that there is an unquestionable need to bring to light issues of gender and “underrepresented” groups (Luk, 2024, p.398), we agree with Van Damme (2023) that the great virtue of *Horizons* is that it opens debates rather than provides definitive answers. As Yılmaz (2023, p.970) suggests, beyond offering an exciting narrative, the book poses an important question: how can we write a coherent global history? This question underlies the most important discussion and the one we consider reflecting on with the most scrutiny.

In what follows, we take a distance from many of the above reviews and their eulogistic tone and lay out our readings of the book. For instance, we believe that Poskett’s narrative strategy of making certain non-Western actors visible also leads to other problems of invisibilization, which become apparent from the moment the author, as Cañizares-Esguerra (2022, p.467) mentions, fails to question “the Eurocentric assumptions that underlie the historiographical categories that are traditionally used to organize the history of science.” Indeed, the new canon of global history, of which Poskett’s book is an example, tends to bring to light only those aspects of the historiography of the non-West that conveniently enlighten the history of the West. We shall deal with this in more detail below.

## Methodology

In his “Introduction,” Poskett warns that trying to cover every country in the world, or every scientific discovery, in his narrative would be foolish and would detract from the enjoyment of the reading. He therefore argues that it is necessary to think of the history of modern science in terms of key moments in global history. That is, rather than a global history of science, as the title of the book indicates, Poskett presents a history of globalization, where science plays a fundamental role in its development and vice versa. Although Poskett achieves his goal and his focus is innovative, his approach based on “major developments” offers a problematic narrative of continuity.

Poskett himself states that he chose four key periods in the transformation of world history to show how the global past shaped modern science. While it is difficult to abandon big pictures for their referential utility, one must be careful when using them because they can exalt periods or promote stories anchored in the careers of great men. Rather than adopting a contextualizing approach that demystifies the genius of individuals, *Horizons* reaffirms the epistemic superiority of these individuals by reaffirming asymmetrical relationships between them and actors from other regions.

Although Poskett includes actors, texts, and places rarely mentioned in traditional histories of science, he presents a diffuse conception of science and the different regions and people that comprise it. Understandably, oversights are to be expected in such an ambitious project, but the problem lies not in the inherent difficulties of studying such a broad temporal or geographical scope. Far from trying to demonstrate a plausible long-term process, such scope hides nuances among the encounters of science. Throughout the book, science is assumed to be a monolithic practice of knowledge production, and differences in the circulation of knowledge amongst societies, geographical spaces, eras, and even within the same Western culture are attenuated. This has ethical implications when it comes to the recognition, or oblivion, of the different expressions of science in diverse societies.

The fact that the book is intended for a broad readership explains some of its spatial and temporal leaps, as well as its tone, which is devoid of discussion. Nevertheless, its omniscient narrative voice compels the reader to learn from the author or even to look at and recognize themself in the variety of otherness described.^3^ Furthermore, the references to the historiography of science are not included to encourage readers to delve into historiographical debates, but to mask anachronisms and expunge omissions, either voluntary or involuntary. This may be a weakness more of global history rather than of the reviewed author, since, as Ghobrial (2019, p.8) has pointed out, “where some regard global history as a forum for writing large-scale synthesis based mainly on secondary literature, other scholars have insisted that global history must preserve a close engagement with philology, local context and, above all, primary sources as its core.” Undoubtedly, despite the approach used, its choice implies giving up either precision or a wide scope and, in both cases, reconfiguring what each individual conceives as globality.

Poskett presents his book as a groundbreaking reinterpretation of the history of science, yet his analysis relies on secondary rather than primary sources. While his approach effectively engages a broad readership, it raises questions about how historical synthesis balances accessibility with scholarly depth. Our further discussion explores how this work builds upon existing contributions in the field and how it could encourage readers to engage more deeply with the cited literature.^4^

Throughout the book, Poskett also provides examples to show how local knowledges transformed modern science. However, due to the formal features of his narrative, knowledge – both material and intangible – from non-Western regions is mostly framed as a contribution to imperial science (and later to Euro-North-American^5^ science) and is denoted in subordinate terms (Delbourgo, 2019, p.383). For example, in chapter 1, “New Worlds,” Poskett recognizes that indigenous people’s knowledge of nature in America challenged old Western assumptions about how scientific knowledge is acquired. A second case, among others, is found in chapter 4, “Economy of Nature,” when he addresses the use of the *Quassia amara* root as a treatment for malaria and characterizes its introduction to Western therapeutics as an event that changed Linnaean classification.

One of the main biases implicit in the narrative is that all the cases presented are selected because they occur under the auspices of European or North American companies or actors. In this sense, the classical Greek texts mentioned in chapter 2, “Heaven and Earth,” are important precisely because they were translations recovered by European culture that led to the Scientific Revolution of the sixteenth and seventeenth centuries. In chapter 4, the only mention made of the medical and naturalistic practices of India is in connection with the more than seven hundred plants collected in the Malabar region between 1678 and 1693 by the colonial administrator and naturalist Henrick van Rheede and reproduced in *The garden of Malabar*.^6^ In chapter 5, “Struggle for Existence,” the paleontological work of Francisco Muñiz and Florentino Ameghino in Argentina gains recognition when linked to Darwin’s work.

At the beginning of each chapter or section, Poskett consistently describes landscapes in detail, in which local actors are seen taking part in scientific experiments, either alone or accompanied by Euro-North-American scientists. Whether it is Moctezuma in his “botanical garden” or Ta’aroa watching the transit of Venus (Poskett, 2022, p.11, 112), to mention just two examples, the images not only create great interest in the book but also idealize local scientific actors. This strategy – useful for attracting readers – is key for arguing that, despite conflicts, knowledge circulated fluently and that, thanks to this, science is a dislocated product that is reformulated with each encounter.

As a result, throughout the book, Poskett takes the existence of modern science for granted and portrays it as something to which the knowledge of non-Euro-North-American actors is added. According to Carrillo Trueba (2006, p.82-99), the Western tradition usually establishes a relationship with other knowledge-generating systems asymmetrically through contempt, idealization, or validation. While contempt – an easily identifiable trait – is something Poskett clearly does not resort to, he does idealize indigenous people as guardians of the harmony between knowledge and nature.

The practice of scientific validation implies the selection of what is considered true, useful, or effective in other knowledge systems and incorporating it into scientific knowledge. In this process, specific practices are atomized and knowledge is filtered. Ultimately, this approach also produces dynamics of extractivism and cultural appropriation, which can be recognized in examples Poskett narrates somewhat superficially. To cite Nieto Olarte (2009, p.14), paraphrasing Arif Dirlik, “the true power of a Eurocentric perspective lies not in the exclusion of ‘the others’, but rather in their inclusion, the inscription of the entire world within a single order and system.”

Poskett implies a definition of “science” based on the exposition of historical examples. While this allows a broader understanding of scientific activity and its multiple expressions, such as knowledge or its practice, it also blurs the differences between these issues and enables him to amalgamate them, regardless of their context. Thus, the book contains multiple expressions of local science that are chosen, filtered, and validated through the logic of Western rationality. Consequently, the narrative flattens and simplifies the “encounters” between knowledge providers and science producers (Turnbull, 1993). In the same sense, this ambiguity reveals remnants of a conception of science where exchanges are shown as reciprocal and seem to be motivated by the sole goal of knowing. Notably, the tension between local histories of science and the supposed universality of knowledge is a key concern in Poskett’s academic work. This suggests that the real challenge lies not in questioning these differences, but in how the history of science is communicated to a wider audience (Poskett, 2024b).

The asymmetry with which Poskett treats non-Euro-North-American science indicates a condescending view. He emphasizes the possession of knowledge or techniques of indigenous people in good faith; however, he loses sight of the local contexts in which they were produced. At first glance, it seems that local communities did not participate in exploitation practices. For example, Poskett (2022, p.12) states that the Mexica expansion throughout the Mexican plateau “was made thanks to the advanced state of Aztec science and technology,” ignoring the fact that their imperial expansion was also based on war with other peoples.^7^ That the author discusses indigenous science without even briefly referring to its respective production contexts reinforces the idea that it took place in idyllic situations. We would suggest that if the science of indigenous actors is to be measured with the same yardstick as that of the West, the practices of exploitation and violence in the former cannot be ignored, when they are recognized in the latter.

This portrayal of science as timeless and apolitical contrasts with Poskett’s own academic stance, where he has emphasized that analytical categories are not ontologically fixed but rather interpretative tools – polysemic and shaped by political perspectives. While one could argue that this discussion is set aside because *Horizons* is a work of science communication, it is still worth reflecting on what intellectual or political position is reinforced by presenting scientific knowledge as decontextualized and detached from historical contingencies.

## Otherness

Throughout the book, Poskett repeatedly mentions that his history is an effort to resist the forces of erasure of non-Western science. To this end, he acknowledges the contributions of scientists from around the world. He attempts to broaden the focus to the global, but this results in a hegemonic narrative that places the United States or Europe at the center. However, he does not problematize why we remember some (Euro-North-American) scientists but not others. The text does not discuss situations that could lead to a denial of non-Western contributions.^8^ Furthermore, due to its intended readership (the general public), Poskett’s book reports on the specialized work of authors who worked the primary sources and retells stories that he refers to as “forgotten,” which leads us to ask: If the stories he wants to retrieve are indeed forgotten, how can they add up to a historiographical body of work that is adequate for such an ambitious science communication project? And also, how does the narrative used invite us or propose for us to remember “the forgotten”?

The exercise of revealing characters and stories to the reader is an extremely attractive narrative tool. Even more so when combined with the goal of recuperating the legacy of those who have been invisibilized. Although it can help readers to remain engaged, that same tool has a negative impact, since it overshadows the works of specialized historical research cited in the text, leaving them out of the argumentative structure.

This reveals that the choice of sources stems from deep-rooted problems in communicating current academic history. While this is not a simple challenge to overcome, the form of writing adopted can mitigate these gaps. A narrative that attempts to take account of all the forgotten actors without addressing the collective construction of historical knowledge only reproduces discourses of invisibility. Conversely, writing that acknowledges the historiographical discussions that have shifted our focus to the so-called peripheries, as well as the partiality and locality of all perspectives, including our own, does a better job of recognizing the individuals who, for metahistoriographical reasons, are generally forgotten.

This discussion serves to help us reflect on the place of “the Other” in the narrative presented by Poskett. Implicitly, the author works with contemporary definitions of countries to refer to historical identities and borders. Even when he intends to problematize the origin of modern science not as a solely European product, but as the result of multiple ideas, cultures, and people around the world, what we read is a narrative that acknowledges the antecedents of science in non-Western regions. Nonetheless, the author claims that such science had to pass through Europe or North America to be validated and, subsequently, spread to the rest of the world to become what we know today.

The book often treats tensions between the local and the global in terms of a national–international duality. Still, the shifting of borders throughout the entire period covered by the book remains largely unmentioned, except in chapter 8, “Genetic States,” where the partition of India and Pakistan in 1947 is addressed. In this and other cases, nationalist characterization reinforces the distinction of otherness. For example, in chapter 3, “Newton’s slaves,” Russia is represented as the “other” because, according to the author, before the seventeenth century, Russia was in a kind of cultural and scientific lethargy. It was not until Peter the Great transformed the country through Newtonian science that it became “a hub of Enlightenment science” and was then integrated into Europe, thus beginning its imperialist history (Poskett, 2022, p.125).

Border changes are by no means a marginal issue. For example, in chapter 6, “Industrial experiments,” several of the mentioned characters go to study at the University of Heidelberg during the mid-nineteenth century in the recently unified Germany. In Poskett’s narrative, nation-states appear to be immovable and ahistorical figures. For instance, territorial consequences are not discussed even when he describes conflicts like the Crimean War. By omitting European expansionist conflicts, Russia and Turkey are situated, once again, “outside” Europe. This includes them in the bag of “otherness,” together with (post)colonial nations, such as Mexico and India, or with other empires, such as the Japanese.

However, no matter how distant or different the nationalities of the scientists portrayed are, the idea of great men is ever present in the book. For example, the navigation of inhabitants of Siberia makes sense as part of the debates fostered by Newton’s physics. Similarly, although Poskett wants to show how the theory of evolution does not arise from Darwin’s proposals, his examples continuously refer to some kind of comparison or connection with the English naturalist (or his context). In chapter 7, “Faster than light,” whether as a revolutionary physicist who inspired young scientists around the globe or as a mentor to some of them, Einstein is a kind of Ariadne’s thread that guides us through the labyrinth of history in Poskett’s narrative.

From the author’s perspective, science is a modernizing force that helps to industrialize and revolutionize nations around the globe. Besides the case of Peter the Great’s Russia, another example is found in chapter 7, where the new theory of relativity and quantum mechanics is associated with the arrival of a better future. Despite this idea, Poskett neither clarifies how particular contexts defined their modernity through science, nor considers the complexities of long periods of time.

The book contains reflections on how non-Western knowledge was disseminated in Europe, which Poskett refers to as co-construction between external and local actors. He treats these supposed exchanges in two ways: either he places them in a horizontality that, on closer inspection, did not exist, or he recognizes violent processes of co-production of knowledge in cases involving the conquest of indigenous societies or the slavery of Afro-descendants. For example, Poskett (2022, p.315, 324, 334) reemphasizes the forgotten cases of genetic development in Mexico, India, and China, while also highlighting the disadvantages of the political system and ideology behind Chinese research into agricultural production.

Poskett intends to be critical by stating that he is not making a triumphant narrative of globalization and by demonstrating that scientific development requires cultural exchanges, in various ways, including those of exploitation. The itinerary proposed by Poskett shows the relationship between European scientific productions in the modern period and their specific relationship with imperial expansion. For this reason, the author emphasizes the role of violence as a transversal axis of power relations between Europeans and the inhabitants of the colonies. This is one of the most relevant points in chapters 3 and 4, where he addresses the relations between empires and eighteenth-century Enlightenment and aims to explain how the system of slave exploitation changed the world of science.

We agree with Poskett’s statements in chapter 4 regarding the fact that the study of natural history cannot be separated from the analysis of global imperial trade. This translates into violent dynamics of knowledge appropriation by Europeans. An instance of this situation took place in Jamaica with the plantation owners Hans Sloane, J. Alexander, and Edward Long, who displayed racist attitudes in their bid to extract samples and botanical data using slaves as informants. Despite the attempt to restore the participation of enslaved Africans and indigenous people to the history of science, instead of recognizing local inhabitants as epistemic authorities and pointing out the conditions that legitimized the production of knowledge within the slave system, the discourse seems to blunt these social and legal frameworks.

In another passage, Poskett (2022, p.105) asserts that without “Indigenous knowledge, Newton’s theories would have remained incomplete” and that “without empire – as well as the dispossession and violence associated with it – there would be no Enlightenment.” Indeed, we are aware that the historiographic exercise demands naming events as they occurred. However, one must be careful how this is done so that the historiographic discourse is not interpreted as a quest for a supposed higher value, like scientific truth (Poskett, 2022, p.2).

Poskett draws attention to the fact that in the great narratives of the history of science, scientists are often presented as great solitary geniuses; he repeatedly states that his book is not such a work. However, he seems to be too reiterative because the story he tells could also be interpreted in this way. Each mention of a non-Euro-North-American scientist is connected with other the stories in great European cities. This is the case with the National Congress of Physics in Paris in 1900, which in chapter 6, “Industrial experiments,” is the central point that brings together physicists from the Ottoman Empire, Russia, India, and Turkey. Their visit to the French capital seems to connect them to the global narrative. The same is true of the University of Heidelberg: after Mendeleev was educated there, he returned to work at the University of St. Petersburg, where he renovated the laboratories following the German model. Another example of this discourse can be found in chapter 8, which, according to the author, presents specific case studies significant to the professionalization of modern genetics, but forgotten in different archives and programs.

Following the same line, one could argue that European advances have been indispensable for developing knowledge and that this knowledge has spread to other countries precisely because of its truth value. Other methodological approaches could further support the point that the author is trying to make. For example, it would be interesting to discuss cases of direct communication between non-European nations, or conceptual and experimental disputes that reveal that true knowledge was not simply created in European cities, but rather that these cities participated in negotiations aimed at reaching a scientific consensus and, by extension, expanding their commercial empires (Azuela, 2018). Furthermore, in Poskett’s narrative, disconnecting from the big cities of Europe implies staying outside the realm of science, like the Russian scientists in 1914 (Poskett, 2022, p.229).

The problem with this generalized view is that the notion of a uniform science reduces its history to flows of knowledge or to the appearance of actors in an extensive narrative. Simplifying the historicity of scientific practices ignores the specificity of the cases. It also underplays the role of actors who have been rendered invisible in knowledge production, such as women, who are often only considered important for their association with men of science. Examples include Sofia Pereiaslavtseva, a Russian scientist and promoter of women’s education in her country, and Maria Rossiiskaia and Ekaterina Wagner, Russian embryologists, who are simply mentioned in one of the book’s arguments (Poskett, 2022, p.195-196). The mere celebration of the fact that women have carried out scientific work, without reflecting on the conditions or contexts in which this occurred, has consequences like the idealization we referred to above. One of the implications is the unintentional or veiled reproduction of an androcentric narrative, in which the history of science remains predominantly masculine, and the participation of other non-hegemonic actors is considered a coincidence.

Including the work done by women does not mean vindicating their work, just as mentioning non-Western actors does not imply doing decolonial work. Although the author has no explicit intention of introducing a gender perspective in his book, recognizing that there are different ways of being culturally, sex-gender, and socially related would enhance the reintroduction of forgotten agents to the history of science.^9^

With this section, we do not intend to advocate for a “denialist” narrative of the centrality of the West over the enterprise of scientific knowledge. Indeed, the dominance of the West or the North Atlantic from the sixteenth century until at least the twentieth century is undeniable. Instead, we must ask ourselves why this region has such weight over knowledge enterprises: What conditions foster such a concentration of knowledge activities? Why are cities like Seville, Paris, London, or Heidelberg indispensable for telling these big-picture stories? We cannot avoid the central role of the West or try to cover it up with discourse about a non-Western story, because if we did, it would remain unquestioned.

## Globality and circulation

Despite his intention to acknowledge that non-Western cultures possess knowledge, from the very first pages Poskett (2022) reveals his Euro-North-American approach in several symptomatic expressions. When he states that “modern science has always depended upon bringing together people and ideas from different cultures around the world” (p.2), it is worth noting that such assemblage is seen as concentrated in a particular region of the world. For the author, although there is knowledge all over the globe, it seems that science is consolidated in the United States and Europe. A case that reflects this vision is when he argues that Copernicus borrowed mathematical techniques from Arabic and Persian texts, which had just been “imported” to Europe back then. Likewise, an encyclopedism masked in simple exchanges, portrayed as totally unidirectional, between actors in the Samarkand region (today Uzbekistan) and the outstanding referents of what we know today as astronomy. Although Poskett claims to vindicate other cultures, his understanding of exchange seems one-sided, where only one party gets to say what is scientific.

Ghobrial (2019, p.8) warns that one of the risks of doing global history is focusing solely on flows and movements of such scale, while ignoring the diversity of processes and contexts that nourish and are influenced by how global processes develop in specific places. Poskett seems to be interested more in depicting the one-dimensional aspects of knowledge circulation. In chapter 7, for example, he states that the Chinese physicist Zhao Zhongya, who worked in California, used the Klein-Nishina formula, developed by a Japanese scientist in Copenhagen, and in doing so “really lead [*sic*] to a more harmonious world” (Poskett, 2022, p.291). But how do these kinds of collaborations lead to world peace? Without a certain degree of naivety, how can we believe this was their purpose?

What’s more, it is implied that if it were not for the fact that these individuals had the opportunity to travel outside their countries, learn German, English, or French, and learn about the discoveries of the West, they would most likely have been left behind in the race for modernity. For example, Poskett claims that Zhou was among many other Chinese physicists who studied abroad in the early decades of the twentieth century and that he was part of “a new generation of Chinese scientists, many of whom made important contributions to the development of modern physics” (Poskett, 2022, p.284). Those involved came from traditional school backgrounds and returned to China as “modern scientists rather than Confucian scholars” (Poskett, 2022, p.287).

It is difficult to demonstrate the existence of knowledge *exchanges* when the only examples cited are concentrated or gathered in places deemed centers in the first place. Even the citations that are supposed to recognize the science of the peripheries end up legitimizing commonplaces in the history of science. Take, for example, chapter 5, where Poskett (2022, p.187-188) states that Argentina’s contribution to the evolutionism of the time was in a sense peripheral, inasmuch as it supplied and enhanced the canon of England, the center where knowledge was produced. He fails to draw attention to the fact that even before their encounters with the West, the different knowledge systems of the world were already engaged in interactions.

It is crucial to transcend the Euro-North-American perspective, which selectively chooses and filters which knowledge it accepts from beyond its boundaries based on its own logic and terms. In this sense, a narrative pattern on the coloniality of power is observed in Poskett (see Quijano, 2014), when he argues that evolutionary ideas of English origin did not represent the beginnings of evolutionary thought: “Before Darwin had even boarded HMS Beagle, people were already discussing the possibility that species might transform” (Poskett, 2022, p.180). He then continues: “From Latin America to East Asia, this chapter uncovers a forgotten history of evolutionary thought, a history which highlights the global origins of the modern biological sciences in an age of conflict” (p.180). Although he does achieve this objective, he is certainly not the first author to do so.^10^ Furthermore, to take four case studies (Argentina, Japan, China, and Russia) as representative of the “global” origins of the biological sciences is arbitrary, since multiplying the number of case studies would equally multiply the number of origins.

Studying the circulation of knowledge suggests that science should be considered a form of communication and scientific knowledge a practice. Such circulation does not only occur in the abstract but is also reflected in material forms of knowledge such as experimental instruments, natural specimens, models, pamphlets, books, drawings, articles, notebooks, and even paintings (Secord, 2004, p.663). This notion of circulation is not passive; on the contrary, it implies an approach that questions what happens when objects/subjects circulate. It also allows us to appreciate other spaces of interchange, considering that they are determined by multiple geographical, social, political, and economic spaces (Silva, Haddad, Raj, 2023, p.9). Thus, whereas we consider that there should be an emphasis on the complexity of scientific exchanges, Poskett’s approach seems to presume that knowledge moves into the United States and Western Europe to become true knowledge and, ultimately, approved scientific practice.

Poskett’s view of circulation can be useful but, as we have noted, problematic. Consequently, we ask ourselves: How can we stop reproducing the power asymmetries from the historical reconstruction of knowledge itself? How can we stop assuming that some local scientific practices opened horizons while others were excluded and forgotten? How can we better understand the power relations in the making-of-knowledge without idealizing the incorporation of local knowledge? As Haraway (1988, p.587) has noted, the idea of infinite vision is an illusion: we can’t aspire to a totalizing vision from everywhere. However, it is precisely our unique positioning that gives us certain responsibilities as historians of the sciences. Thus, how we understand the concept of “circulation” means taking a stance on how knowledge is created and what we believe true knowledge to be.

## Final considerations

In *Horizons*, Poskett’s intentions are bold and illuminating in making visible those who have been rendered invisible in the history of science. He pursues this goal by dismantling a notion of science that he seems to assume his readers have. However, the way he structures his chosen examples tends to decontextualize non-Western knowledge. Pointing out the existence of other actors, his examples are certainly informative and can be interpreted as a genuine effort to make a more global history of science. Nonetheless, the text ends up reproducing, even if involuntarily, a traditional and universalizing Euro-North-American vision. Moreover, his narrative of knowledge production, which draws from so many regions, cultures, and contexts, assumes that they share the same vision of science. Escaping from this universalist notion requires keeping in mind that science is a social practice – that is, understanding its internal logic(s) and its political, social, institutional, and historical specificities (Barahona, Raj, 2022, p.3). Although Poskett addresses the social character of the scientific enterprise, the way he points out the flows of science emphasizes their dimension as true knowledge and a product resulting uniquely from the Euro-North-American validation of other knowledge systems.

But, more importantly, in the epilogue, Poskett shows a wary interest in China and in the fact that its investment in science surpassed that of the United States in the last years of the twentieth century, as he does in Kenya and Ghana’s development of artificial intelligence. He holds similar opinions about space science and climate change in non-Euro-North-American regions: all topics that he describes, not without suspicion, in the context of what he calls the “New Cold War.” He argues that this framework is shaping the scientific research of our time and that a balance between globalization and nationalism must be found. Although Poskett (2022, p.367-368) approves of the fact that the Asian and Middle Eastern regions “are capable of producing science,” he claims that the governments of these regions are private companies that collect personal data and invest in expensive special programs whose purpose or value is far from clear. In a globalized world, why should it be strange for countries outside the North Atlantic to develop science when its ethical use should be a matter of general interest? This is a question that arises from reading his narrative, and we think it lacks an answer. One way or another, it does tend to reinforce the idea that although modern science is an accumulation of non-Western knowledge added to Euro-North-American knowledge, it can only be responsibly used in the hands of Europeans and North Americans.

Throughout the book, science is shown as a Western cultural phenomenon that moves across eras from a local geographic space (specifically Europe and North America) to a global one, with contributions by individuals from diverse origins. This narrative is full of general historiographic commonplaces: the purity and beauty of American nature before colonization; classical mathematics and astronomy recovered by the Arabs in the fifteenth century; Newton’s theory of universal gravitation; the exotic astronomy of the Muslims, the Chinese, and the Indians; Polynesian islanders’ sailing practices; the theory of evolution proposed by Darwin; Einstein’s theory of relativity; contemporary genetics; and so forth. Despite trying to refute the idea of genius ascribed to the aforementioned scientists, the emphasis remains on great discoveries, the Nobel Prize, and aspirational stories of the poor and the dispossessed who manage to become recognized scientists despite not coming from the West.

Poskett’s global history offers a continuous expansion – in time – of the Scientific Revolution of the sixteenth and seventeenth centuries and a unidirectional flow across exchange networks in a world that was about to become globalized. However, it is essential to note that the concept of the Scientific Revolution began its academic life after the Pyrrhic victory of Western Europe in the Second World War, giving way to Eurocentrism in the history of science (Raj, 2017, p.447) in the first years of the Cold War, as a result of its cultural diffusion (Secord, 2023). This is not only a historiographical position, but a political and circular one: which agents we consider or not depends on how valuable their knowledge is according to “our” definition of science.

In the analysis of Poskett’s text, we can observe that, although he intends to offer a global history of science, his referential framework is constructed from a Western-centric viewpoint, namely, that of the emergence of Western civilization. In addition, his argument is articulated in terms that resemble the “rise of the West” perspective of William H. McNeill (1963). According to Adelman (2 Mar. 2017), such approaches tend to include diverse hegemonic regions, which are considered centers of knowledge, to illustrate and explain the development of the West. Moreover, by focusing on nation-state relations as the unit of historical analysis, they often focus on actors considered nation-builders. This tendency is also evident in *Horizons*, where Poskett attempts to distance himself from the traditional heroes of the Scientific Revolution, yet ends up reinforcing established narratives around naturalists and scientists recognized in the history of science.

It is crucial that the global history of science, as an academic discipline, transcends narratives centered on the nation-state and its major scientific figures. Rather, it should focus on a broader framework of exchanges and encounters encompassing multiple origin points and meanings within a global background. In other words, the task is to elaborate “moving histories” as opposed to the Euro-North-American position, to question simplistic ideas of discoveries and knowledge diffusion, as well as the associated practices and technologies. It is equally important to identify the factors that influence the success or failure of this circulation in specific contexts. Although there is no single dominant method in this area, there is consensus on the goal of countering Euro-North-American narratives (Ghobrial, 2019, p.11; Adelman, 2 Mar. 2017; Gavrus, 2016, p.363).

As we have already noted, another aspect that deserves attention is the way Poskett portrays the circulation of knowledge in *Horizons*. In more recent texts about the global history of science, Poskett (2024a, 2024b) has suggested adopting the concept of “friction” to emphasize the consequences of unequal power dynamics, as well as the concept of scale as an analytical and actor’s category, with its political, spatial, and historical dimensions, to avoid replicating the political dynamics of contemporary globalization. Nonetheless, this process of circulation involves a dynamism that affects not only forms of knowledge, but also their contexts and meanings. Knowledge circulation is not a linear, fluid, or uniform process. It is related with complex interactions between different actors who reinterpret and re-signify science (Raj, 2021, p.199-204; Silva, Raj, 2016; Espinosa, 2015). Therefore, the concept of circulation in the global history of sciences invites us to consider not only the movement, but also the multiple layers of meaning that are generated along its trajectory, which allows us to appreciate its richness and complexity across time and space (Ghobrial, 2019; Conrad, 2016; Raj, 2017).

Moreover, it is crucial to recognize that all narratives are inherently selective and influenced by both what is included and what is omitted. Constructing a comprehensive global history of science means doing more than merely incorporating actors or case studies from different regions of the world. However, *Horizons* offers a series of preponderantly unidirectional exchanges, prioritizing centripetal movement, i.e. “towards the center,” over site-specific contextualization. Its approach risks leading, and in fact does lead, to narratives that are flat in terms of networks and suggest a kind of global predestination. These narratives simplify the complexity of interactions between societies of different cultural paradigms and, moreover, may neglect local particularities that are fundamental to a richer and more nuanced understanding of knowledge (Adelman, 2 Mar. 2017). Focusing exclusively on circulation as mere movement, then, risks losing sight of how diverse cultures and contexts reconfigure and shape the meaning of what moves.

Faced with these challenges, several questions arise. For example: How can we counter the Euro-North-American approach that only integrates data from other regional, cultural, and temporal traditions into its own (scientific) culture? How can we link the general with the particular, or the local with the global, considering that different cultures have approached these issues in different ways? One possible way could be through the perspective of microhistory: from the analysis of specific details, it is possible to challenge the teleology and triumphalism that recurrently infiltrate global history. Nowadays, interest in the relationship between microhistory and global history has manifested itself in the publication of a rich and varied collection of works, which are increasingly grouped under the concept of “global microhistory.” This new but growing trend reflects an effort to broaden the scope of historical study, integrating local perspectives into a wider framework. In doing so, it seeks to generate dialogue between different traditions and approaches, thus enriching the understanding of global history while at the same time being sensitive to the asymmetries of power (Ghobrial, 2019, p.10).

Two important points at the intersection of the micro and the global are relevant here: the agency of individuals or groups, and their entanglements. The agency of nonhuman actors must be framed at different scales. This is necessary so that these global micro-stories address particular (local) cases situated in larger (transnational or global) contexts. To summarize, the connections of individuals at large scales must converge. This convergence can influence global histories, their dynamics, the circulation of human and nonhuman actors, and even new knowledge produced in the process (Berg, 2023; Skotnes-Brown, Silva, 2025). It can demonstrate some of the multiple traditions of knowledge that contribute to the production of scientific knowledge in particular times and places. When analyzing the numerous cross-cultural influences or the socially and globally diverse worlds surrounding various scientific practices, it is crucial to emphasize contingencies and acknowledge both successes and failures in our historical reconstructions. This approach enables us to explain the origins and development of our current practices and the outcomes we have achieved (Easterby-Smith, 2019). By addressing these issues, it opens a field of study that also challenges the limitations of traditional approaches, offering new perspectives that help to better understand the complexity of scientific knowledge in an interconnected world.

*Horizons* appears at an opportune time in the field of historiography to reflect on how the global history of science can or should be written. However, a problem with this approach is that the ability to access sources from anywhere in the world through libraries, newspaper archives, and digital repositories means searches are limited to previously studied areas of research. This prioritizes what can be known about these areas, overlooking the reasons why these repositories have such sources, whether primary or secondary (Putnam, 2016, p.377).

As we mentioned at the beginning of this paper, reading *Horizons* has helped us carry out an analytical exercise on the scope and limitations of the history of science from a global perspective. Indeed, we do not have a final answer on how this should be done, nor do we intend to have one. Rather, we seek to continue the discussion on the various theoretical and methodological problems involved in undertaking such historiographic exercises (Adelman, 2 Mar. 2017; Raj, 2017; Conrad, 2016; Fan, 2012; Hodges, 2011). Of course, we agree with Poskett that doing global history does not mean covering all spaces and times. Such an undertaking would be practically impossible. However, nor should it consist in trying to include as many places in the world as possible, as if this could make an approach global. Global history should not be confused with “planetary” history. Regarding our respective otherness, we think that if more emphasis was placed on asymmetrical power relations, diffusionist narratives could be avoided, and clarity could be gained regarding the communities that have produced their own histories and the reasons why we may access them. This is, without a doubt, a sign of the globality in which we live, and which is also projected in what we write.

Although this essay resulted from our reading of *Horizons*, we would also like it to be a starting point for debating the challenges of global history and reflecting on their possible implications: What gives a narrative globality? How can we carry out methodologically situated research? How can we construct a global history that respects all the otherness we do not share?

Just like the supposed forgotten scientists who participated in the scientific fairs of the nineteenth century, it seems that non-Euro-North-American actors and authors are integrated into global narratives to the extent that they participate in the science communication dynamics established by the old protagonists of this story. Thus, the global circulation, discussion, and coproduction of science is not only a historical question but also a current problem for science communication. Despite the growth of academic communities in non-Euro-North-American regions of the globe, there needs to be a rapprochement, in every possible sense, between all identities and alterities. That is why we are here, writing in a language that isn’t our own, to participate in discussions about the scope of global history. However, “here we are” is also a manifestation of our existence and our place in this globalized world – and yes, here we are.

## Data Availability

Not deposited in a data repository.

## NOTES

1 Organized by the Seminario Universitario de Historia, Filosofía y Estudios de las Ciencias y la Medicina, at National Autonomous University of Mexico (Universidad Nacional Autónoma de México), and coordinated by Ana Barahona in collaboration with Kapil Raj.

2 Here and elsewhere, citations of works in languages other than English were translated by the authors.

3 Unlike a voice that assumes it can account for the complexity of the global world, a statement of individuals’ limitations in achieving such a view warns readers about the situational, theoretical, and methodological limitations of each writing exercise, for example, see Iggers, Wang, Mukherjee (2008, p.1-18).

4 In his academic work, Poskett has himself historicized efforts to move beyond a solely European history of science. This suggests that the discussion should center on how best to disseminate these perspectives.

5 This is a reference to Conrad’s (2016, p.54) notion of “Euro-American,” to which we add “North,” because it is contrary to world historiography, which is based on the idea that scientific development has occurred in the regions of the United States and Western Europe in isolation from the rest of the world.

6 Originally published in Latin as *Hortus Indicus Malabaricus*, *The garden of Malabar* – the term adopted by Poskett – is an English translation that misrepresents the object of the work, emphasizing the “garden” as a physical space rather than focusing on its true purpose of inventorying the local flora (Raj, 2017, p.455-457).

7 The “Mexica militarism” implemented by the Triple Alliance has been widely studied and is considered by various authors to be one of the most powerful in the pre-Columbian world in ancient Mesoamerica. See Bueno Bravo (2012).

8 In his work *Silencing the past*, Trouillot (1995) considers that precisely one of the causes of these “forgettings” is that historical production implies the unequal access of groups and individuals to the means to establish their narratives as true.

9 The gender perspective enables the analysis of different phenomena of reality, considering the implications and effects of the social relations of power between genders (Serret-Bravo, 2008).

10 Three classic studies on this phenomenon are Gould (2002) and Bowler (1983, 1988).
